# Species-independent contribution of ZBP1/DAI/DLM-1-triggered necroptosis in host defense against HSV1

**DOI:** 10.1038/s41419-018-0868-3

**Published:** 2018-07-26

**Authors:** Hongyan Guo, Ryan P. Gilley, Amanda Fisher, Rebecca Lane, Vanessa J. Landsteiner, Katherine B. Ragan, Cole M. Dovey, Jan E. Carette, Jason W. Upton, Edward S. Mocarski, William J. Kaiser

**Affiliations:** 1 Department of Microbiology, Immunology, and Molecular Genetics, University of Texas Heath San Antonio, San Antonio, TX 78229 USA; 2 Department of Molecular Biosciences, LaMontagne Center for Infectious Disease, Institute for Cellular and Molecular Biology, University of Texas at Austin, Austin, TX 78712 USA; 3 Department of Microbiology and Immunology, Stanford University School of Medicine, Stanford, CA 94305 USA; 4 Department of Microbiology and Immunology, Emory Vaccine Center, Emory University School of Medicine, Atlanta, GA 30322 USA

## Abstract

Necroptosis complements apoptosis as a host defense pathway to stop virus infection. Herpes simplex virus shows a propensity to trigger necroptosis of mouse cells and mice even though cell death is blocked in human cells through UL39-encoded ICP6. This ribonucleotide reductase large subunit (R1) nucleates RHIM-dependent oligomerization of RIP3 kinase (RIPK3, also known as RIP3) in mouse cells but inhibits activation in cells from the natural human host. By interrogating the comparative behavior of ICP6-deficient viruses in mouse and human cells, here we unveil virus-induced necroptosis mediated by Z-DNA-binding protein 1 (ZBP1, also known as DAI). ZBP1 acts as a pathogen sensor to detect nascent RNA transcripts rather than input viral DNA or viral DNA generated through replication. Consistent with the implicated role of virus-induced necroptosis in restricting infection, viral pathogenesis is restored in *Zbp1*^−/−^, *Ripk3*^*−/−*^ and *Mlkl*^−/−^ mice. Thus, in addition to direct activation of RIPK3 via ICP6, HSV1 infection in mice and mouse cells triggers virus-induced necroptosis through ZBP1. Importantly, virus-induced necroptosis is also induced in human HT-29 cells by ICP6 mutant viruses; however, ZBP1 levels must be elevated for this pathway to be active. Thus, our studies reveal a common, species-independent role of this nucleic acid sensor to detect the presence of this virus. HSV1 ICP6 functions as a bona fide RHIM signaling inhibitor to block virus-induced necroptosis in its natural host. Altogether, ZBP1-dependent restriction of herpesvirus infection emerges as a potent antiviral armament of the innate immune system.

## Introduction

Programmed necrosis, or necroptosis, has been recognized for contributions to antiviral host defense and inflammatory tissue damage. This pathway is mediated by receptor-interacting protein (RIP) kinase (RIPK)3-dependent phosphorylation of mixed-lineage kinase domain-like (MLKL), a pseudokinase^1–4^. Necroptosis is an alternate outcome to apoptosis triggered by caspase compromise. RIPK3 activation occurs via one of three different RIP homotypic interaction motif (RHIM)-containing adaptors common to humans and mice: (i) RIPK1; (ii) TIR-domain-containing adapter-inducing interferon (IFN) (TRIF); or (iii) Z-nucleic acid (NA)-binding protein 1 (ZBP1, also known as DAI or DLM-1). Activation of RIPK3 results in the recruitment and phosphorylation of MLKL into a necrosome complex that translocates to the plasma membrane^4–6^ and drives membrane permeabilization^7–10^. Necroptotic death cuts short replication and restricts dissemination of murine cytomegalovirus (MCMV) in its natural mouse host^11–13^. Importantly, UL39-encoded ICP6, the large subunit of ribonucleotide reductase (R1) protein of herpes simplex virus (HSV)1 and 2, like the MCMV M45-encoded R1 sequence homolog, blocks RHIM-dependent pro-necrotic signal transduction in cells from its natural human host^13,14^. ZBP1 was implicated as a pathogen sensor capable of recognizing HSV1 DNA in mouse cells^15^, along with cGAS and STING^16–18^. ZBP1 functions as crucial adaptor for RHIM-dependent activation of RIPK3-dependent necroptosis during MCMV infection^11,12,19,20^ and has more recently been implicated in the induction of necroptosis by influenza^21–23^ as well as vaccinia virus (VV)^24^. Evidence is accumulating to suggest that ZBP1 senses accumulation of RNA rather than DNA to initiate RHIM exposure and recruitment/activation of the RIP kinases^12,21–23^. It remains unclear how this potential pathogen sensor plays into the natural function of RHIM-containing inhibitors of necroptosis in HSV1 infection^14^.

Viruses vest heavily in sustaining cell viability until they produce progeny, disseminate, and transmit to new hosts. Large DNA viruses have evolved to encode a range of gene products that suppress cell death^25–27^ to prevent the elimination of infected cells in addition to modulating other aspects of host defense. Necroptosis is unleashed as an alternate to apoptosis, most critically when caspase-8 activity is compromised by virus-encoded caspase-8 inhibitors^1,28,29^, possibly contributing to the emergence of necroptosis as a host defense pathway in mammals^30^. VV-infected cells resist necroptosis via viral suppression of ZBP1 recognition of NA but remain sensitive to tumor necrosis factor (TNF)-induced necroptosis dependent on the virus-encoded caspase inhibitor^24,29^ and host cell expression of RIPK3^1^ to activate MLKL. MCMV provided the first evidence for virus-induced necroptosis independent of TNF where the M45-encoded viral inhibitor of RIP activation prevents induction of this pathway by blocking ZBP1-dependent oligomerization and activation of RIPK3^11,12^. M45 mutant MCMV is attenuated in wild-type (WT) mice, but viral replication and pathogenesis is normalized in either ZBP1-deficient or RIPK3-deficient mice^11,13^. During natural infection, the RHIM-dependent association of ZBP1 and RIPK3 is blocked by MCMV M45, thereby avoiding premature cell death and supporting the production of progeny virus in support of dissemination and pathogenesis.

ZBP1 is an IFN-inducible protein that binds double-stranded Z-form DNA and RNA^31,32^. Although implicated as a sensor of cytosolic DNA with the potential to trigger an IFN response^15^, any central role as a DNA sensor has long been questioned^18^. ZBP1 contains tandem amino-terminal Z-NA-binding domains, Zα1 and Zα2 (also called Zβ)^33^. In addition to Z-NA-binding domains, ZBP1 signaling employs RHIM signaling elements, at least two of which are functional^30^. ZBP1 RHIM-A-dependent binding to RIPK3 drives necroptosis as well as activation of nuclear factor-κB (NF-κB)^34,35^. Both RHIM-A and RHIM-B engage RIPK1 to activate NF-κB. Recently, the perinatal lethality of RIPK1-RHIM mutant knock-in mice was shown to be dependent on ZBP1^36,37^, although ZBP1 signaling requirements in this setting are yet to be determined. Nevertheless, ZBP1 is predicted to act as a homeostatic harbor for RHIM-containing RIP kinases where RHIM-dependent functions of RIPK1 restrain ZBP1-mediated activation of RIPK3. In the context of virus infection, the amino-terminal Zα1/Zα2 domains of ZBP1 have been shown to sense the accumulation of RNA transcripts to initiate RHIM exposure and recruitment/activation of the RIP kinases^12,21–23^. During MCMV infection, the Zα2 domain senses viral transcription^12,19^. Seasonal influenza A virus (IAV), a segmented, negative-strand RNA virus, relies on ZBP1 Zα2 to sense viral RNA^21–23^; whereas, pandemic IAV suppresses necroptosis^38^ in a pattern that suggests this virus encodes an inhibitor. In contrast to MCMV and IAV, VV-induced necroptosis requires the Zα1 domain for necroptosis induction^24^, suggesting alternate mechanisms governing ZBP1 recognition of NA depending on the particular virus. Additionally, ZBP1 has been shown to inhibit HSV1 replication independent of nucleic acid sensing via regulation of ICP0^39^.

HSV ICP6 blocks TNF-induced necroptosis in human cells^14^, contrasting markedly the ability of this same viral protein to trigger necroptosis in mouse cells^40,41^,^42^. The species-specificity of ICP6 function suggested a role as a host restriction factor in the non-natural mouse host; however, RIPK3 kinase-dependent necroptosis also occurs with ICP6-deficient virus^20^. In this study, we have elaborated a distinct mechanism through which HSV1 induces necroptosis in addition to the direct activation of RIPK3 via RHIM interactions in mouse cells^40,41^. We find that ICP6 RHIM mutant HSV1 triggers virus-induced ZBP1/RIPK3/MLKL-dependent necroptosis in both mouse and human cells, thereby establishing the role of ZBP1 as an evolutionarily conserved sensor of HSV1 in both species. Our observations expand the number of examples where necroptosis is induced via this Z-NA sensor. Striking parallels emerge between MCMV and HSV1, with the RHIM-dependent suppressor of necroptosis from either virus functioning to suppress ZBP1-dependent death. Importantly, HSV1 transcription is required for initiation of necroptosis independent of input viral DNA or replication-dependent amplification of viral DNA. Furthermore, the increased susceptibility of ZBP1-deficient mice to ICP6 mutant virus clearly demonstrates an antiviral role for ZBP1 independent of other, species-specific characteristics of this virus. Taken together, our findings expand the relevance of ZBP1 as a sensor of herpesvirus infection in mammals.

## Methods

### Cells and viruses

Primary mouse embryonic fibroblasts (MEFs) were generated from E13.5 embryos and used within the first five passages. SVEC4-10, NIH3T3, 3T3-SA, MEF, 293T, and HT-29 cells were cultured in Dulbecco’s modified Eagle medium containing 4.5 g/ml glucose, 10% fetal bovine serum (Atlanta Biologicals), 2 mM l-glutamine, 100 U/ml penicillin, and 100 U/ml streptomycin (Invitrogen). The ICP6-null mutant HSV1 ΔICP6 and its parental strain KOS were propagated and titered as previously described^14^.The ICP6 RHIM mutant HSV1 F*mut*RHIM and its parental strain F were kindly provided by Dr. Jiahuai Han (Xia Men University, Xiamen, China). F and F*mut*RHIM viral stocks were propagated and tittered on monolayer cultures of Vero cells. MCMV K181 and MCMV-M45mutRHIMviruses were generated as described^13^.

### Reagents and immunoblotting

Dimethyl sulfoxide, actinomycin D (ActD), and phosphonoformate (PFA) were purchased from Sigma. Murine TNF-alpha and caspase inhibitor zVAD-fmk were purchased from Pepro Tech and Enzo Life Sciences, respectively. GSK’840^43^ from GlaxoSmithKline has been described before. MLKL inhibitor necrosulfonamide (NSA) was purchased from Calbiochem. Immunoblotting and immunoprecipitation (IP) were performed as previously described^13^. For non-reducing gel analysis, lysates were prepared in the absence of dithiothreitol. The following antibodies were used in cell death assays, immunoblot (IB), and IP analyses: mouse anti-mouse IFNα/β receptor 1 antibody (clone MAR1-5A3, BD Pharmingen); mouse anti-ZBP1 (clone Zippy-1, AG-20B-0010); rabbit anti-MLKL (phospho S345) (ab196436, Abcam); rat anti-MLKL (MABC604, Millipore); mouse anti-ICP0 (sc-53070, Santa Cruz); mouse anti-FLAG (clone M2, Sigma); anti-myc (clone 9E10, Santa Cruz Biotech); mouse anti-β-actin (clone AC-74, Sigma-Aldrich); rabbit anti-RIPK3 (2283, ProSci); and goat anti-RIPK3 (sc-47364, Santa Cruz).

### Generation of ZBP1 knockout SVEC4-10 cells

Endogenous ZBP1 of SVEC4-10 cells was targeted for knockout using CRISPR/Cas9 strategy^44^. Briefly, sgRNAs were identified and designed with online bioinformatics tools (crispr.mit.edu and e-crisp.org). A target site was selected corresponding to the 29th amino acid of ZBP1 located in exon 2. The sgRNA was cloned into AflII linearized gRNA_Cloning Vector (a gift from George Church (Addgene plasmid # 406 41824)) by Gibson assembly. The cloned gRNA, hCas9 Vector (a gift from George 407 Church (Addgene plasmid # 41815)), and pQCXIP (Clontech) vector were co-transfected into SVEC4-10 cells, and cells selected with 2 μg/ml puromycin 24-h post transfection. Following outgrowth, single cells were subcloned by limiting dilution, and clonal populations surveyed for absence of ZBP1 expression by immunoblotting.

### Plasmids and transduction

To generate human ZBP1 (hZBP1)-expressing lentiviral vector, 3XFLAG epitope-tagged hZBP1 open reading was inserted into retroviral expression vector pQCXIH (Clontech). Murine ZBP1 (mZBP1) and mutants inserted in pQCXIH were described previously^22^. All plasmids were verified by DNA sequencing. Retrovirus stock preparation and transduction were described previously^14^. Reconstituted HT-29 or SVEC4-10 cell lines were selected in 500 or 400 μg/ml hygromycin, respectively.

### Cell viability assays

Determination of intracellular ATP levels were performed as previously described^14^ using the Cell Titer-Glo Luminescent Cell Viability Assay kit (Promega) on a Synergy HT Multi-Detection microplate reader (Bio-Tek). An alternative cell viability assay was used to monitor cell permeability using the live cell impermeant nucleic acid stain-Sytox Green (Invitrogen) with a Citation Cell Imaging Multi Mode Reader (Bio-tek). Cells (10^4^ cells/well) were seeded into Corning 96-well tissue culture plates. Sixteen to eighteen hours post seeding, medium was replaced with 50 µl of viral inoculum. After infection for 1 h, cells were cultured in the medium containing 50 nM Sytox Green and 1.25 μg/ml Hoechst 33342 (Thermo Fisher Scientific). Two images of each well were collected. Sytox Green-positive and Hoechst-positive cells per image were counted. The level of cell death was determined by calculating the number of Sytox Green-positive cells relative to total cells determined by Hoechst 33342-positive cell number.

### Animal model of HSV1 infection

Mice were housed in facilities at the University of Texas Health Science Center at San Antonio (UTHSCSA). C57BL/6J mice (WT), *Dai*^−/−^^45^, *Ripk3*^−/−^^46^, and *Mlkl*^−/−^^47^ mice at 6–8 weeks of age were infected with HSV1(F*mut*RHIM) with 1 × 10^7^ plaque-forming units per mouse by intraperitoneal (i.p.) injection. Three days post infection, spleens were excised and used to prepare homogenates, which were then titered by plaque assay. All animal experiments were performed in accordance with protocols by the Institutional Animal Care and Use Committee at UTHSCSA.

### Statistical analyses

Data of cell viability or death are represented as the mean ± SD. All experiments were repeated at least three times with similar results. One-way analysis of variance with Dunnett’s multiple comparison post-test analyses were conducted using GraphPad Prism 5. *p* < 0.05 was considered significant.

## Results

### HSV1 infection triggers ZBP1-dependent necroptosis in mouse cells independent of ICP6

**Fig. 1:**
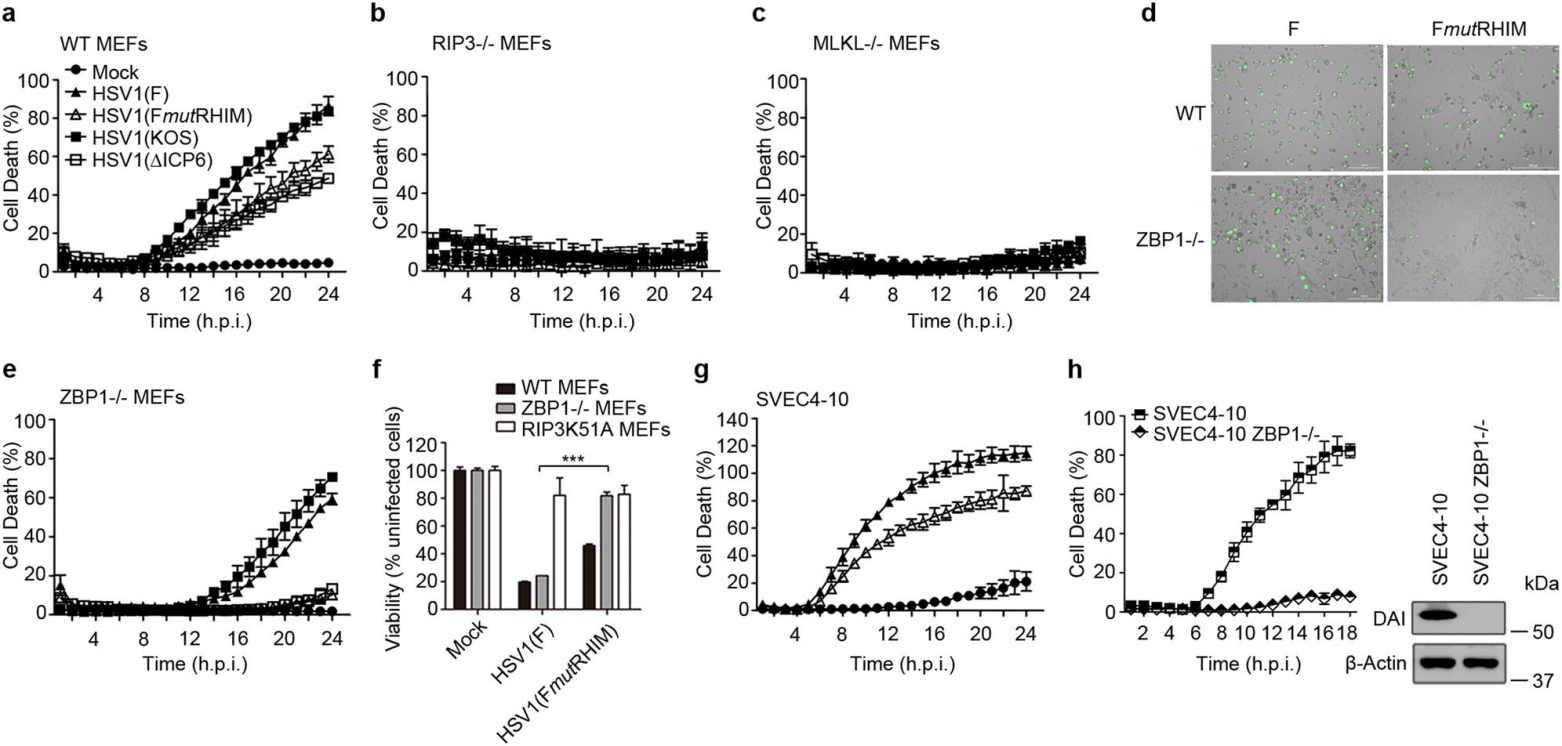
HSV1 infection induces ZBP1-dependent but ICP6-independent necroptosis in mouse cells. **a**–**c** Kinetics of cell death of WT MEFs (**a**), *Ripk3*^−/−^ MEFs (**b**), and *Mlkl*^*−*/−^ MEFs (**c**) infected with HSV1 (F or KOS) or HSV1 mutant virus, respectively, at a multiplicity of infection, measured in real time by Sytox Green incorporation. **d** Micrograph images of WT MEFs and *Zbp1*^*−/−*^ MEFs infected with HSV1 (F) or HSV1 (F*mut*RHIM) for 24 h in the presence of Sytox Green. Scale bar = 200 μm. **e** Kinetics of cell death of *Zbp1*^−/−^ MEFs infected with HSV1 (F or KOS) or HSV1 ICP6 mutant viruses, respectively, measured in real time by Sytox Green incorporation. **f** Cell viability of WT, *Zbp1*^−^^/−^, and RIPK3 kinase inactive (RIPK3K51A) MEFs infected with HSV1 (F) or HSV1 (F*mut*RHIM). **g** Kinetics of cell death of SVEC4-10 cells infected with HSV1 (F) or HSV1 (F*mut*RHIM), measured in real time by Sytox Green incorporation. **h** Kinetics of cell death of ZBP1-deficient SVEC4-10 cells following infection with HSV1 (F) or HSV1 (F*mut*RHIM). ZBP1 expression was confirmed by IB (right). Black solid triangle represents F, black solid square represents KOS, hollow triangle represents F*mut*RHIM, hollow square represents ∆ICP6, and solid circle represents Mock. hpi hours post infection. See also Supplemental Fig. 1

Previous reports^40,41^ revealed the importance of ICP6-dependent RIPK3-MLKL necroptosis in mouse cells and mice. We observed RIPK3-dependent death of mouse cells infected with ICP6 mutant virus^20^. In order to compare overall patterns of cell death, we employed MEFs and observed cell death that was completely independent of ICP6 or its RHIM. Death induced by either ICP6-null (∆ICP6) or RHIM mutant (F*mut*RHIM) viruses proceeded with slower kinetics than with WT viruses (Fig. 1a). Infection with WT or mutant virus led to a dramatic increase in necrotic cell death as monitored by time-lapse imaging (Supplemental Movie 1 and 2). Whether death was induced by WT or ICP6 mutant HSV1, RIPK3 and MLKL were required (Fig. 1b, c), consistent with known parameters of necroptosis^20^.

To further characterize the genetic requirements for initiation of ICP6-independent death, we evaluated *Zbp1*^*−/−*^ MEFs (Fig. 1c–f). As previously reported^40,41^, mutant cells succumbed to WT virus even though we found *Zbp1*^*−/−*^ MEFs to resist ICP6 mutant virus-induced death. Also, as reported^20^, RIPK3 (K51A) kinase dead knock-in MEFs resisted death induced by either WT or mutant virus. In contrast, RIPK1 (K45A) kinase dead knock-in MEFs and *Trif*^*−/−*^ MEFs remained sensitive to WT or mutant virus-induced death (Supplementary Fig. 1a-c), albeit with slower kinetics than observed with C57BL/6 MEFs (Fig. 1a). This ICP6-independent virus-induced death depended on the cellular RHIM-containing proteins, ZBP1 and RIPK3, but not TRIF or the kinase activity of RIPK1. To confirm the contribution of ZBP1 to this cell death, we evaluated ZBP1-deficient SVEC4-10 endothelial cells (Fig.1g, h)^12^. Similar to the pattern in MEFs, ZBP1-deficient SVEC4-10 cells resisted death. Thus, HSV1 ICP6 mutant virus triggers necroptosis via ZBP1, a pattern reminiscent of MCMV M45 mutant virus^11,13^.

### ZBP1 restricts HSV1 ICP6 mutant virus replication in vitro or in vivo

**Fig. 2:**
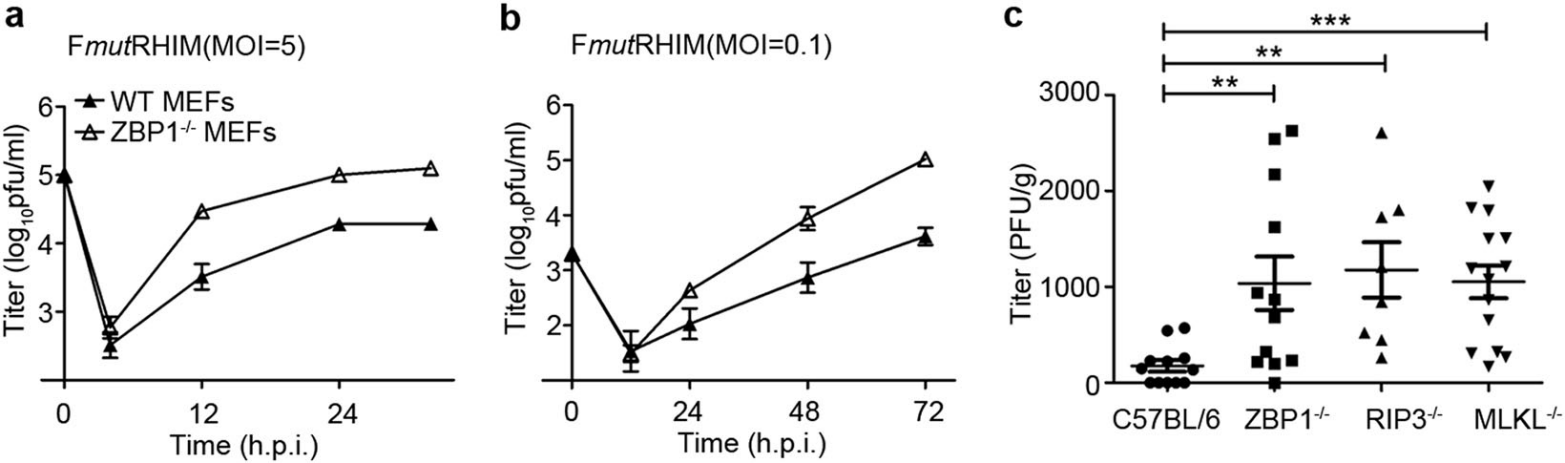
HSV1 (F*mut*RHIM) virus attenuation in vitro or in vivo is reversed when ZBP1 is absent. **a**, **b** Viral titers of WT MEF and *Zbp1*^−/−^ MEF cells infected with HSV1(F*mut*RHIM) viruses at (**a**) MOI = 5 or (**b**) MOI = 0.1 for indicated times. Cells together with the supernatants were harvested and titered by a standard viral plaque assay. pfu plaque-forming units. **c** Viral titers of spleen of WT, *Zbp1*^−/^^−^, *Ripk3*^−/−^, and *Mlkl*^−/−^ mice infected with 1 × 10^7^ pfu HSV1 (FmutRHIM) per mouse via intraperitoneal inoculation (i.p.). One-way ANOVA multiple comparison post-test analyses were conducted using GraphPad Prism 5. *p* < 0.05 was considered significant

To investigate the impact of ZBP1-dependent cell death on HSV1 infection, WT or *Zbp1*^*−/−*^ MEFs were infected with HSV1 viruses to generate single-step (multiplicity of infection; MOI = 5) or multi-step (MOI = 0.1) growth curves (Fig. 2a, b). HSV1 mutant virus replicated robustly, with >10-fold higher titers in *Zbp1*^*−/−*^ MEFs relative to WT MEFs. Thus, ZBP1 restricts HSV1 replication in mouse cells when the RHIM of ICP6 is compromised. To further address the contribution of ZBP1 to host defense in vivo, WT, *Zbp1*^*−/−*^, *Ripk3*^−/−^, or *Mlkl*^*−/−*^ mice were inoculated via i.p. injection with HSV1 ICP6 RHIM mutant virus. As shown in Fig. 2c, at 3 dpi viral titers in the spleen were significantly elevated in *Zbp1*^*−/−*^, *Ripk3*^−/−^, or *Mlkl*^*−/−*^ mice compared to WT mice. Altogether, HSV1 RHIM mutant virus induces ZBP1-dependent necroptosis and limits viral replication both in cells and in mice, albeit, not to the levels or restriction observed for MCMV replication.

### ZBP1 recruits RIPK3 to mediate necroptosis induced by HSV1 ICP6 mutant virus

**Fig. 3:**
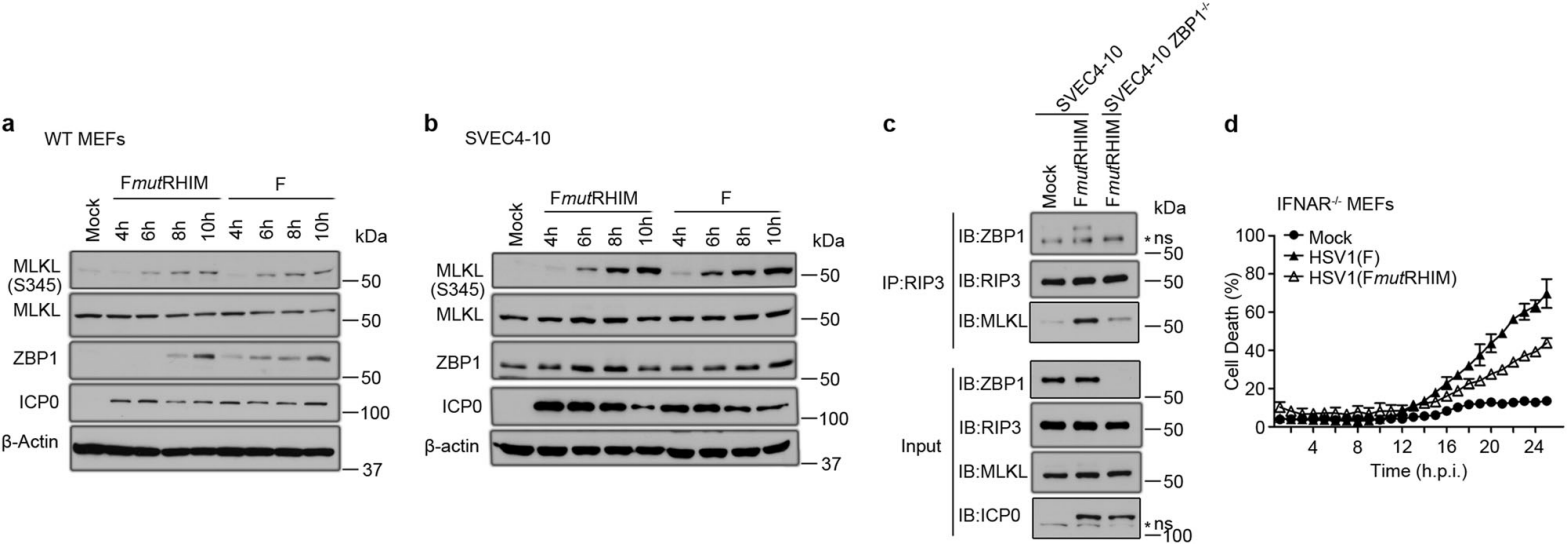
HSV1 (F*mut*RHIM) infection activates MLKL and drives ZBP1-RIPK3 complex formation. **a**, **b** IB analysis to detect p-MLKL, total MLKL, ZBP1, ICP0, and β-actin from WT MEFs (**a**) or SVEC4-10 (**b**) infected with HSV1 (F) or HSV1 (F*mut*RHIM) for the indicated times. **c** Immunoprecipitation (IP) analysis to detect the interaction of ZBP1 and RIPK3 in SVEC4-10 cells. Wild-type SVEC4-10 cells were infected with HSV1 (F*mut*RHIM), mock infected cells as the control. ZBP1-deficient SVEC4-10 cells infected with the same amount HSV1 (F*mut*RHIM) were also employed as a control. RIPK3 were immunoprecipitated, and ZBP1, RIPK3, and MLKL were analyzed by IB. Whole-cell extract (5% input) was examined in parallel for RIPK3, ZBP1, MLKL, and HSV1 proteins-ICP0. Black solid triangle represents F, hollow triangle represents F*mut*RHIM, and solid circular represents mock. **d** Kinetics of cell death of type I interferon receptor (IFNAR)-deficient MEFs infected with HSV1 (F) or HSV1 (F*mut*RHIM). See also Supplementary Figure 2

We next evaluated the kinetics of MLKL phosphorylation, a requisite step preceding the induction of plasma membrane permeability during necroptosis. Both WT and mutant virus triggered MLKL phosphorylation (p-MLKL) as early as 6 h after infection (Fig. 3a), 4 h before cells became permeable (Fig. 1a). We also evaluated MLKL phosphorylation in SVEC4-10 and 3T3-SA cells (Fig. 3b and Supplementary Fig. 2a), which appeared with similar kinetics despite the elevated basal level of ZBP1 expression in SVEC4-10 and 3T3-SA relative to MEFs. We next sought to characterize whether a ZBP1/RIPK3 necrosome-like complex was formed during infection. As shown in Fig. 3c, both RIPK3 and MLKL coimmunoprecipitated ZBP1 in extracts of infected SVEC4-10 cells. Thus, HSV1 ICP6 RHIM mutant virus infection promotes the rapid assembly of a ZBP1-RIPK3-MLKL necrosome-like complex that drives RIPK3-dependent phosphorylation of MLKL and subsequent death of virus-infected cells.

ZBP1 is both inducible by and an inducer of IFN. For IAV-induced necroptosis, both RIG-I as well as type-1 IFN receptor signaling contribute to upregulation of ZBP1 and induction of IAV-induced death^21,23^. ZBP1 is normally expressed at low levels in MEFs and showed the expected increase in WT MEFs after infection with either WT or mutant HSV1 at a high MOI (Fig. 3a). This pattern is similar to the induction of ZBP1 following MCMV infection^11^. Type I IFN receptor-deficient MEFs (*Ifnar−/−*) remained sensitive to HSV1-induced necroptosis, even though a reduced level of cell death was observed (Fig. 3d and Supplementary Fig. 2b). Thus, IFN signaling is not necessary for ZBP1-induced necroptosis but may influence efficiency of cell killing given the dramatic impact of this cytokine on ZBP1 in IAV- and VACV-induced death^21–23^. Treatment of SVEC4-10 cells with increasing concentrations of IFNAR-neutralizing antibody did not reduce levels of virus-induced programmed necrosis (Supplementary Fig. 2c) suggesting that, analogous to MCMV infection^11^, the basal level of ZBP1 in these cells was sufficient to support necroptosis.

### Requirements for induction of ICP6 mutant virus-induced necroptosis

**Fig. 4:**
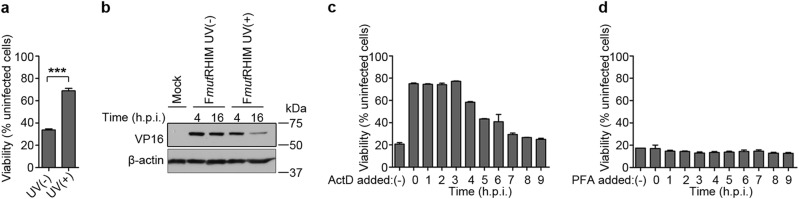
HSV1 (F*mut*RHIM)-induced necroptosis requires nascent transcription. **a** Cell viability of SVEC4-10 cells infected with HSV1 (F*mut*RHIM) (UV (−)) or UV-irradiated F*mut*RHIM virus (UV (+)). Data with error bars depict mean ± standard deviation (SD); with significance ***p < 0.001 indicated above bars. **b** IB of VP16 and β-actin in SVEC4-10 cells infected by F*mut*RHIM or UV-irradiated virus at 4 and 16 hpi. **c**, **d** Cell viability of SVEC4-10 cells infected with HSV1 (F*mut*RHIM) as well as treated with actinomycin D (ActD) (**c**) or phosphonoformate (PFA) (**d**) at the indicated times post infection

MCMV-induced necroptosis requires viral transcription^12,19^, and, recently, evidence with influenza suggested that viral RNP complexes promote ZBP1 activation^21–23^. To identify steps during HSV1 infection necessary for ZBP1 activation, we initially evaluated the capacity of ultraviolet (UV)-treated ICP6 mutant virus to induce necroptosis (Fig. 4a). VP16, an abundant late phosphoprotein packaged into virions, was detectable at similar levels at 4 hours post infection (hpi), confirming similar input was used (Fig. 4b). VP16 levels declined dramatically by 16 hpi with UV-inactivated mutant virus, indicating the expected impact of inactivation on replication. UV-inactivated mutant virus failed to induce robust necroptosis, indicating that virion components such as DNA were insufficient to trigger necroptosis. We next evaluated transcription and viral DNA replication inhibitors. Initially, we assessed the role of newly generated transcripts by employing ActD, a potent inhibitor of RNA synthesis. As shown in Fig. 4c, addition of ActD completely blocked HSV1 ICP6 mutant virus-induced necroptosis, even when added up to 3 hpi. The protective effect of ActD declined when added at 4 hpi or later. These findings demonstrate that HSV1-induced necroptosis requires RNA synthesized at early times following infection. We next assessed the role of viral DNA amplification in virus-induced necroptosis by employing phosphonoformic acid (PFA), a potent inhibitor of herpesviral DNA polymerases. As shown in Fig. 4d, PFA treatment did not inhibit virus-induced necroptosis. HSV1 encodes immediate-early, early (E), and late (L) transcripts^48^. As PFA prevents DNA synthesis as well as “true” L phase gene expression, the signal driving ZBP1 activation occurs temporally during E or “leaky” late viral gene expression, a period of active symmetric transcription of the viral genome. In summary, these results indicate that virus-induced necroptosis is triggered following virus entry and virion transport to the nucleus, requires nascent transcription, but proceeds independent of viral DNA replication.

### Virus-induced necroptosis requires the Zα2 and RHIM-A domain of ZBP1

**Fig. 5:**
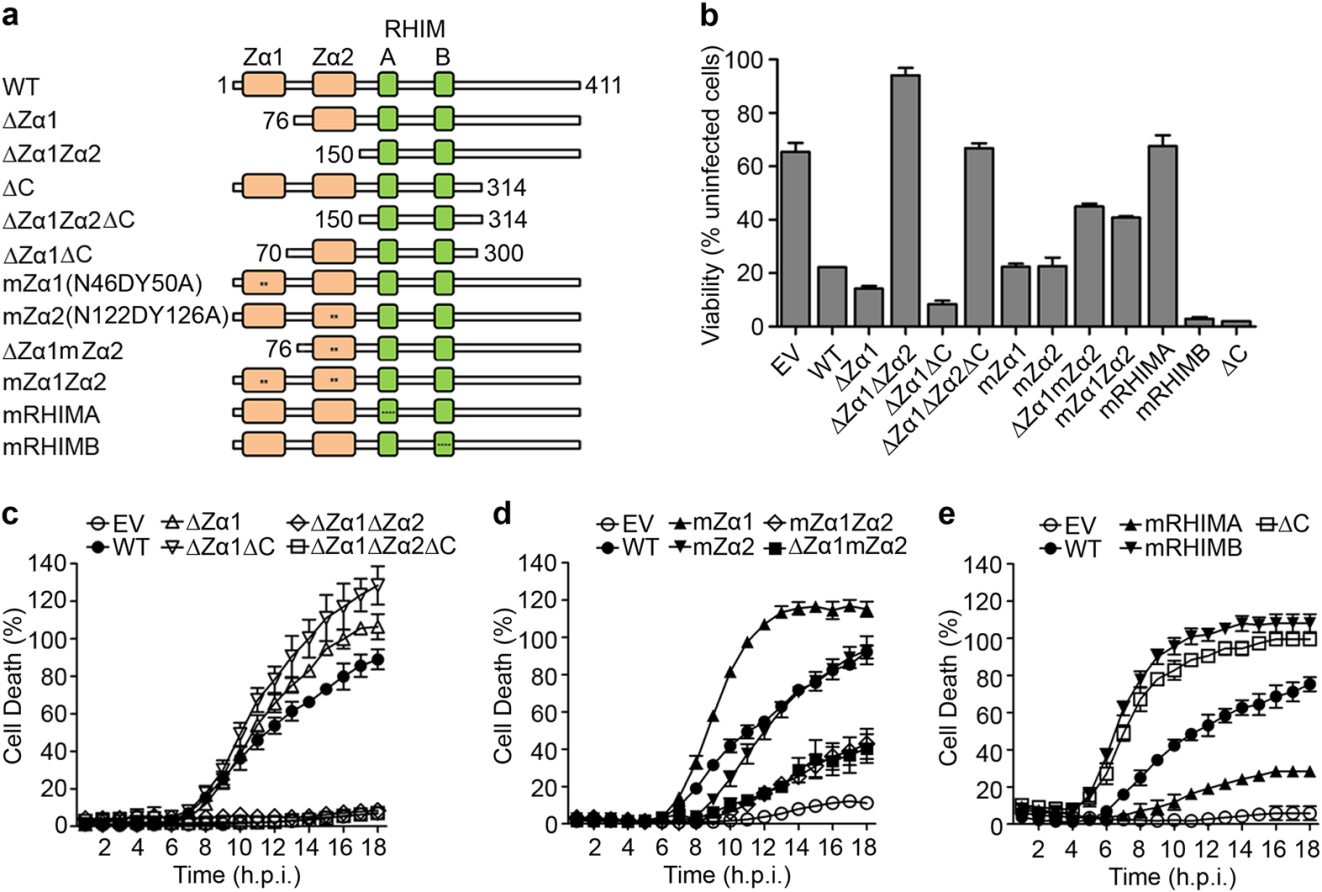
HSV1 (F*mut*RHIM)-induced necroptosis requires ZBP1’s nucleic acid-binding domain as well as RHIM-A domain. **a** Schematic representation of the murine ZBP1 mutants. Red rectangle depicts Z-nucleic acid-binding domains, green rectangles represent RHIM-A and -B. Asterisk represents amino-acid mutation localized in Zα1, Zα2, RHIM-A, and RHIM-B. Numbers indicate amino-acid positions. Names of the mutants are listed on the left. **b** Cell viability of ZBP1-deficient SVEC4-10 cells reconstituted with indicated ZBP1 mutant constructs infected with HSV1 (F*mut*RHIM). **c**–**e** Kinetics of cell death of indicated ZBP1-reconstituted cell lines infected with HSV1 (F*mut*RHIM), measured in real time by Sytox green incorporation. See also Figure S3

To determine the regions of ZBP1 required for necroptosis induction by ICP6 mutant virus, we generated a series of ZBP1 mutants with three tandem N-terminal FLAG tags (Fig. 5a), reconstituted SVEC4-10 ZBP1-deficient cells with mutant forms of ZBP1, and then analyzed the susceptibility of these cell to virus-induced necroptosis. Protein expression was confirmed by IB (Supplementary Fig. 3a). Reconstituted cell lines were infected with HSV1. As shown in Fig. 5b and Supplementary Fig. 3b, expression of WT ZBP1 restored sensitivity to virus-induced necroptosis, while control empty vector (EV) reconstituted cells remained resistant. To assess the contribution of the Z-NA-binding domains, we generated a series of deletion mutants in ZBP1. Zα1 (∆Zα1) was dispensable for virus-induced necroptosis and this mutant showed accelerated the kinetics of death (Fig. 5a–c). When both Z-NA-binding domains, Zα1 and Zα2, were deleted (∆Zα1Zα2), necroptosis was abolished, indicating a requirement for the Zα2 domain for susceptibility to virus-induced necroptosis. A similar pattern was observed with MCMV^12^.

We next assessed the importance of Z-NA-binding residues^33^, N46 and Y50 in the Zα1 domain and N122 and Y126 in the Zα2 domain. Introduction of point mutations in the Zα1 domain (N46D and Y50A; mZα1) enhanced cell killing (Fig. 5d) comparable to the Zα1 deletion (Fig. 5c). Virus-induced necroptosis was as efficient in cells expressing the Zα2 (N122D and Y126D; mZα2) mutant as WT ZBP1-expressing cells (Fig. 5d). Combining point mutations in both domains (mZα1mZα2) or the Zα1 deletion with the point Zα2 mutants N122D and Y126D (∆Zα1mZα2) led to a stark compromise in cell killing relative to either WT or mZα2 (Fig. 5d). Of note, Zα2 N122 and Y126 have been previously shown to be crucial for IAV-induced necroptosis^22^ indicating similarities in requirements for ZBP1 activation by IAV and HSV1. Both the Zα1 and Zα2 domains likely cooperate for ZBP1 activation during HSV1 infection, with the Zα2 alone being sufficient for necroptosis. Diminished but detectable levels of cell killing were sustained in cells expressing either mZα1mZα2 or ∆Zα1mZα2 indicating additional residues in the Zα domains contribute to ZBP1 recognition during HSV1 infection.

ZBP1 RHIM domains are crucial for binding to RIPK3 and RIPK1. As anticipated from prior studies on MCMV and IAV, cells expressing the ZBP1 RHIM-A (mRA) were completely resistant to necroptosis induced by HSV1 (Fig. 5e) supporting that this domain is sufficient to mediate ZBP1-RIPK3 interaction to initiate necroptosis induced by MCMV^11^, IAV^22^, and, here, HSV1. Mutation of RHIM-B or C-terminal deletion (∆C) displayed even more rapid kinetics of necroptosis than WT ZBP1 suggesting that these regions regulate activation of RIPK3. This seems reminiscent of the rapid cell killing observed for the Zα mutants ∆Zα1 and mZα1. The potential toxicity of these ZBP1 mutants may have contributed to our failure to generate cells that express only the Zα2 and RHIM-A regions of ZBP1. Taken together, these results suggest that NA-binding of the tandem Zα domains of ZBP1 enable RHIM-A exposure and binding to RIPK3 to execute necroptosis, whereas the C-terminal region of ZBP1 encompassing the RHIM-B domain temper necroptotic signaling.

### Virus-induced necroptosis in human cells

**Fig. 6:**
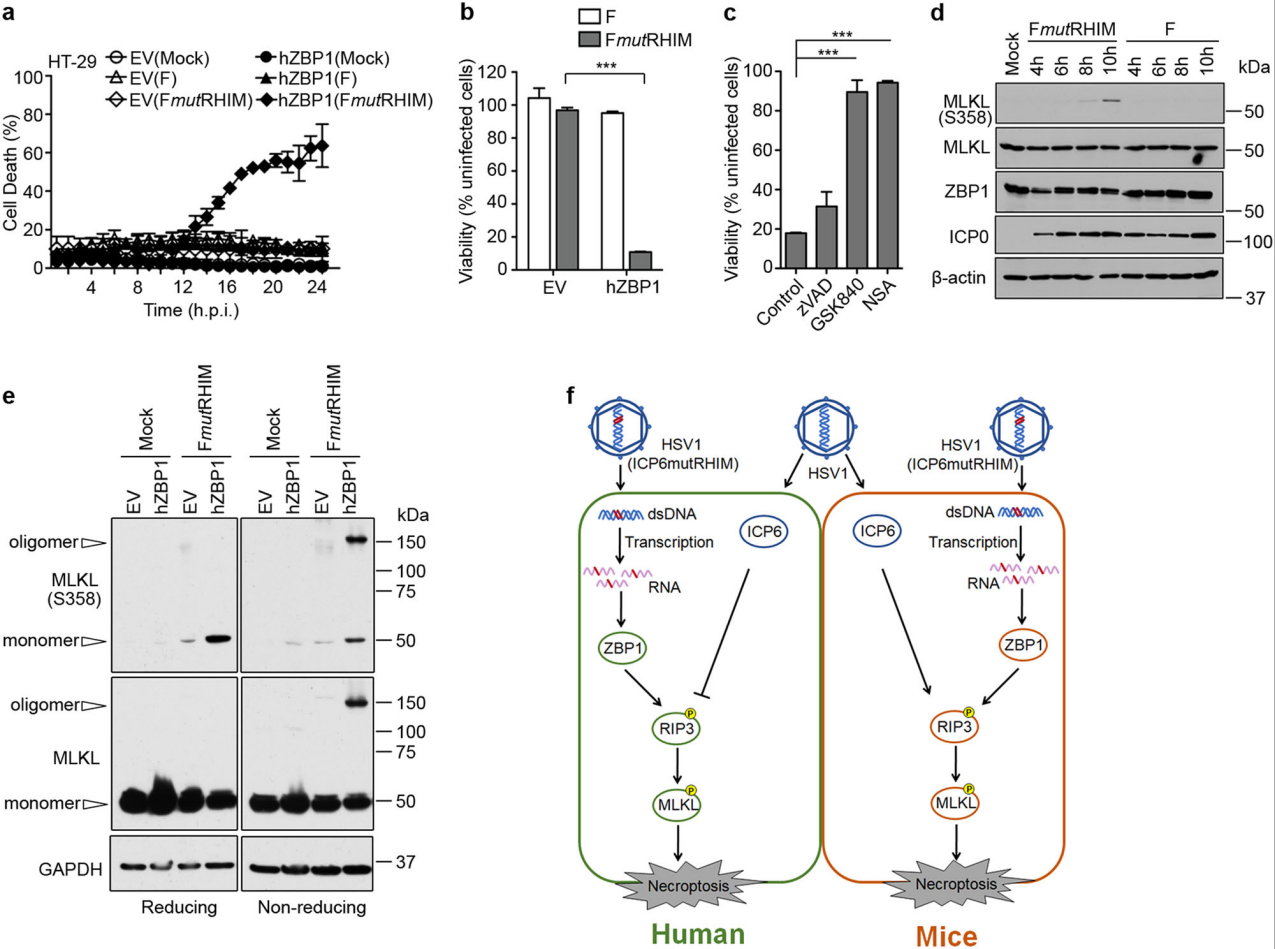
HSV1 (F*mut*RHIM) drives necroptosis in human cells reconstituted with ZBP1. **a** Kinetics of cell death of HT-29 cells with or without human ZBP1 (hZBP1) expression infected with HSV1 (F) or HSV1 (F*mut*RHIM), measured in real time by Sytox Green incorporation. **b** Cell viability of HT-29 cells with or without hZBP1 expression infected with HSV1 (F) or HSV1 (F*mut*RHIM). Data with error bars depict mean ± standard deviation (SD); with significance ***p < 0.001 indicated above bars. **c** Cell viability of HT-29 cells expressing hZBP1 infected with F*mut*RHIM in the absence or presence of zVAD (25 μM), GSK840 (5 μM), or NSA (1 μM). Data with error bars depict mean ± standard deviation (SD); with significance and ***p < 0.001 indicated above bars. **d** IB analysis to detect p-MLKL, total MLKL, hZBP1, ICP0, and β-actin from HT-29 cells with or without hZBP1 expression infected with HSV1 (F) or HSV1 (F*mut*RHIM) for the indicated times. **e** IB analysis to detect MLKL oligomers and monomers from EV or ZBP1-expressing HT-29 cells following infection with F*mut*RHIM for 16 h by SDS-PAGE under reducing or non-reducing conditions. **f** Schematic of HSV1 and HSV1 (ICP6mutRHIM) virus infection in mouse and human cells

We have previously shown that HSV1 ICP6 RHIM function prevents TNF-induced necroptosis in human cells^14^. To evaluate the potential contribution of ZBP1 to initiation of necroptosis in human cells, we enhanced the expression of ZBP1 in necroptosis-sensitive human HT-29 cells, a cell line that lacks ZBP1, but expresses RIPK3. HT-29 cells are permissive for HSV1 infection^2,14^. ICP6 RHIM mutant virus induced cell death in ZBP1-expressing HT-29 cells but not in ZBP1-deficient control cells (Fig. 6a, b). This cell death was blocked by the RIPK3 inhibitor GSK840 or the MLKL inhibitor NSA, but not by the pan-caspase inhibitor zVAD, consistent with a pattern of RIPK3/MLKL-mediated necroptosis triggered by ZBP1 (Fig. 6c). MLKL phosphorylation (Fig. 6d) and oligomerization (Fig. 6e) were detectable as early as 8 hpi with ICP6 RHIM mutant virus with cell death appearing by 12 hpi. In contrast, WT HSV1 did not induce necroptosis in either EV control or ZBP1-expressing cells. Therefore, HSV1 requires the RHIM domain in ICP6 to prevent ZBP1-induced necroptosis in cells from its natural host, signaling that is reminiscent of MCMV infection in mice^11^. Both of these herpes viruses employ RHIM antagonism to block antiviral necroptosis. ZBP1 signaling emerges as a common target of disruption for α- and β-herpes viruses in their respective natural hosts.

## Discussion

Necroptosis has the potential to eliminate virus-infected cells, limiting replication, and restricting spread. In human cells, HSV1-encoded ICP6 is a RHIM signaling suppressor that blocks TNF-induced necroptosis^14^; whereas, in mouse cells, this RHIM recruits RIPK1 and RIPK3 to drive necroptosis^14,40,41^. Our current study sought to determine how necroptosis proceeds in mouse cells independent of ICP6 RHIM signaling^20^. To identify the molecular basis for this cell death, we monitored HSV1 RHIM mutant-induced necroptosis in fibroblasts that carried mutations in components of the known necroptotic pathways. As anticipated, the common mediators of necroptosis, MLKL and RIPK3, were essential for death. Loss of ZBP1 also conferred complete protection, revealing this sensor as the crucial factor initiating ICP6-independent HSV1-induced necroptosis (Fig. 6f). ZBP1 binds RIPK3, triggering recruitment of MLKL to form a virus-induced necrosome-like complex that promotes MLKL phosphorylation within 6 hpi, leading to necroptosis in most cells by 16 h. Furthermore, we observed higher titers of HSV1 RHIM mutant virus in ZBP1-deficient cells and mice, indicating the host defense potential of necroptosis in limiting HSV1 replication.

ZBP1 emerges as a potent initiator of necroptosis following viral infection with HSV1 (this study), MCMV^11,12^, vaccinia^24^, and influenza^11,22,23^. ZBP1 was initially characterized for its ability to bind double-stranded Z-form DNA or RNA^31,33,49^. The current evidence from influenza, vaccinia, MCMV, and now HSV1 that transcripts accumulating during infection are the most likely PAMP that interacts with ZBP1 under natural conditions. Striking parallels emerge between comparison of MCMV RHIM mutant- and HSV1 RHIM mutant-induced necroptosis. Deletion of both the Zα1/2 domains or combined mutation of known nucleic acid coordinating residues in both domains dramatically reduced cell killing for both herpes viruses supporting the proposed sensor role of ZBP1 in surveillance of viral pathogens (this study, refs ^12,19^). Of note, only mutation of the Zα2 domain was necessary to eliminate ZBP1 activation following influenza infection indicated different requirements for influenza and herpes viruses. For either herpesvirus, viral genome deposition following entry or viral replication did not activate ZBP1; however, inhibition of transcription at early times resulted in a near-complete block in necroptosis. Collectively, these findings support ZBP1 activation by specific cellular or viral RNAs induced following infection. While it is possible that RNAs serve as template for proteins that activate ZBP1, evidence suggest that Zα domains interact with newly synthesized influenza and MCMV RNA, both of which have secondary structure giving double-stranded character^19,22^. No specific ZBP1 activating RNA has yet been identified and the RNA–protein interactions may promote ZBP1 recognition as has been suggested for the influenza viral RNP^21^.

ZBP1 is both a highly IFN-inducible protein^32^ and a potent activator of IFN^15^. Following influenza infection, the RIG-I pathway drives IFN production that in turn elevates ZBP1 to levels sufficient to support necroptosis^21^. Consistent with IFN contributing to an increase in ZBP1, we also note elevation of ZBP1 in primary fibroblasts following HSV1 infection; however, where IFN is essential for influenza-induced necroptosis, elimination of IFNAR signaling did not prevent ZBP1-induced necroptosis in the context of herpesvirus infection. Furthermore, in cell lines with sufficient ZBP1 expression, antagonizing IFN signaling did not impact necroptosis, similar to the behavior of MCMV RHIM mutant virus, which is normalized in the absence of ZBP1 but remains completely attenuated in IFNAR-deficient mice^13^. Thus, in apparent contrast to both influenza and vaccinia, neither HSV1 nor MCMV requires IFN signaling to provide sufficient sensor for the initiation of necroptosis.

How does ZBP1 activation occur only in infected cells despite the apparent promiscuity of the Zα1/Zα2 domains in binding nucleic acid given ZBP1 binds endogenous RNAs in uninfected cells^19^? Recently, several groups independently characterized a perinatal pattern of embryonically lethality in RIPK1^RHIM/RHIM^ mutant mice that could be completely rescued by the elimination of ZBP1^36,37^. One interpretation of this unexpected phenotype is that RIPK1 functions as a RHIM-dependent repressor of ZBP1 activation in uninfected cells. By impeding RHIM signaling, both MCMV and HSV1 control the RHIM-dependent unleashing of ZBP1 function and activation RIPK3. In this study, we find that deletion of the C terminus of ZBP1 or mutation of the RHIM-B augments the kinetics HSV1-induced necroptosis, thus the RHIM-B tempers the efficiency of ZBP1 killing. As RIPK1 binds to either the RHIM-A and RHIM-B domains of ZBP1, it is tempting to speculate that the pro-death manifestation of the RHIM-B mutant is due to the lack of repression by the RIP1-FADD-Casp8/cFLIP_L_ axis^34,35^ and suggests at minimum additional factors likely dampen ZBP1 signaling in uninfected cells.

In summary, this study defines a ZBP1-directed antiviral necroptosis that eliminates HSV1-infected cells and restricts viral replication in vitro and in vivo. During infection of its natural host, HSV1 employs the RHIM in ICP6 to prevent formation of a virus-induced ZBP1/RIPK3/MLKL necrosome-like complex in addition to the TNF-induced necrosome^14^. Thus, both MCMV and HSV1, as well as their close rodent and primate relatives, employ similar strategies to sustain cell viability during infection. This highlights the selective pressure to maintain subversion despite evolutionary divergence in overall pathogenesis. Altogether, ZBP1 emerges a critical and specific sensor of infection by many mammalian viruses.

## Electronic supplementary material

Fig S1

Fig S2

Fig S3

Sup movie 1

Sup movie 2

Supplementary figure legends
