# Supplementary figures and images for: Species-independent contribution of ZBP1/DAI/DLM-1-triggered necroptosis in host defense against HSV1
Source: Cell Death Dis. 2018 Jul 26;9(8):816. doi: 10.1038/s41419-018-0868-3 (PMC6062522; doi:10.1038/s41419-018-0868-3)

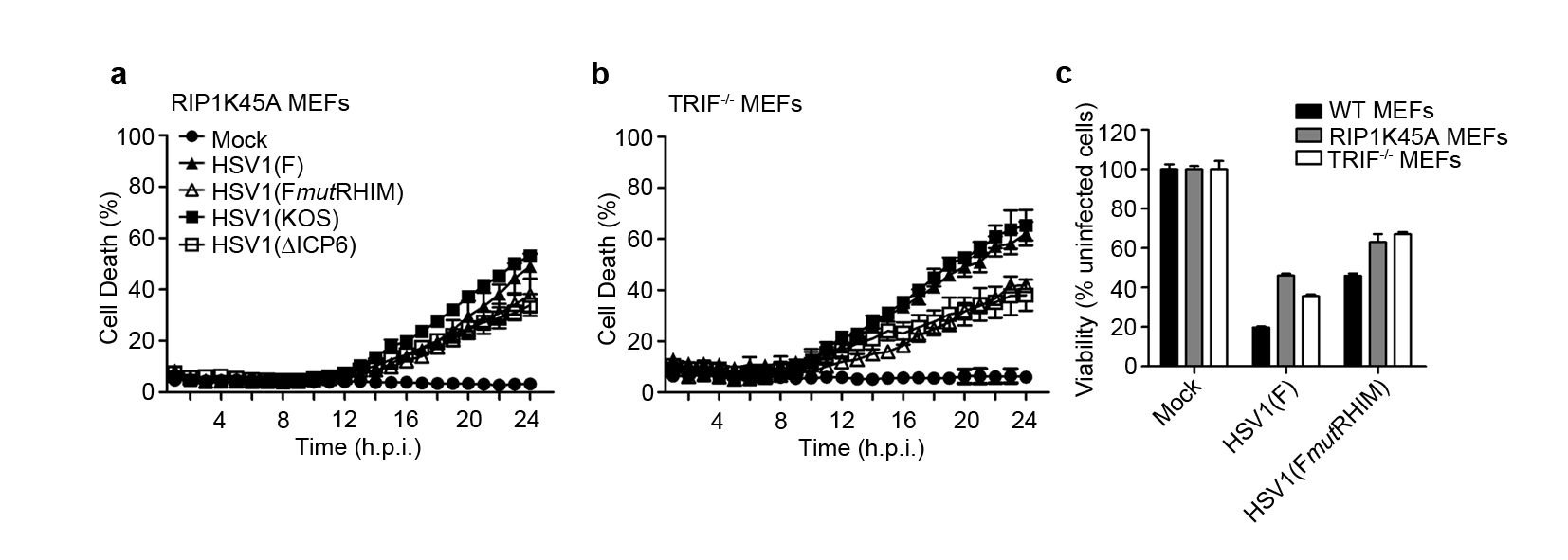

Supplement: Supplementary file 1 — Fig S1 [file 41419_2018_868_MOESM1_ESM.tif]

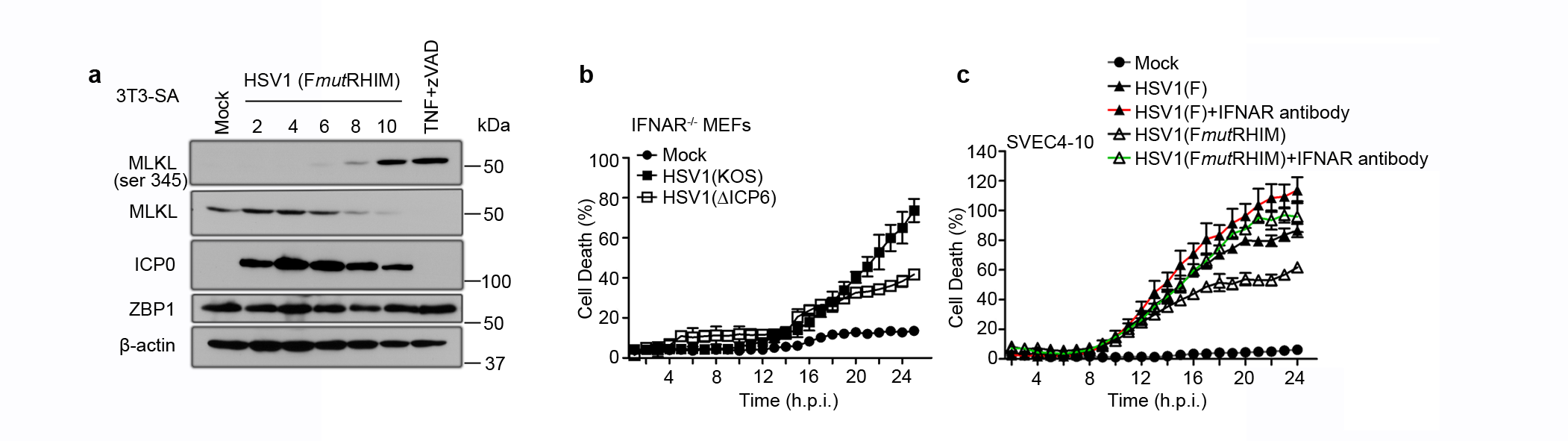

Supplement: Supplementary file 2 — Fig S2 [file 41419_2018_868_MOESM2_ESM.tif]

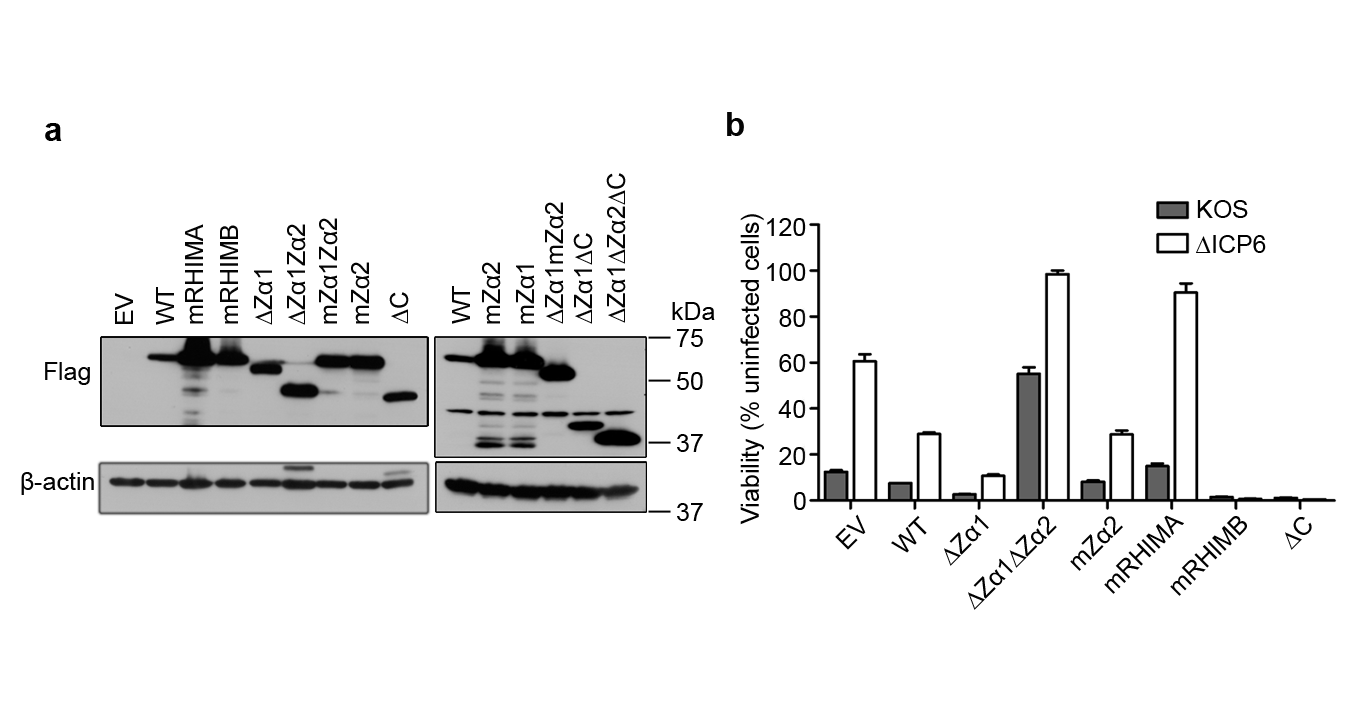

Supplement: Supplementary file 3 — Fig S3 [file 41419_2018_868_MOESM3_ESM.tif]
