# Supplementary material for: Species-independent contribution of ZBP1/DAI/DLM-1-triggered necroptosis in host defense against HSV1
Source: Cell Death Dis. 2018 Jul 26;9(8):816. doi: 10.1038/s41419-018-0868-3 (PMC6062522; doi:10.1038/s41419-018-0868-3)
Supplement: Supplementary file 6 — Supplementary figure legends [file 41419_2018_868_MOESM6_ESM.docx]

**Supplemental Figures**

**Supplementary Fig. 1**

***a* and *b*.** Kinetics of cell death of RIP1 kinase inactive (RIP1K45A) MEFs (A) and *Trif*^-/-^ MEFs (B) infected with HSV1(F or KOS) or mutants (F*mut*RHIM or ∆ICP6, respectively), measured in real time by Sytox Green incorporation.

***c*.** Viability of WT MEFs, RIP1K45A MEFs and *Trif*^-/-^ MEFs infected with HSV1 (F) or HSV1 (F*mut*RHIM).

***d*.** Micrograph images of WT MEFs and *Zbp1*^-/-^ MEFs infected with HSV1 (F) or HSV1(F*mut*RHIM) for 2 h in the presence of SytoxGreen. Scale bar=200 μm.

**Supplementary Fig. 2**

***a*.** IB analysis to detect p-MLKL, total MLKL, ZBP1, ICP0 and β-actin from 3T3-SA cells infected with HSV1 (F*mut*RHIM) for the indicated times. As a positive control, cells were treated with TNF in combination with zVAD for 3 h.

***b*.** Kinetics of cell death of SVEC4-10 cells infected with HSV1 (F) or HSV1 (F*mut*RHIM) in the absence or presence of type I interferon receptor (IFNAR) neutralizing antibody.

**Supplementary Fig.3**

***a*.** IB analysis to detect ZBP1 expression in SVEC4-10 deficient cells reconstituted with the WT or mutant forms of ZBP1.

***b*.** Cell viability of cells reconstituted with the indicated ZBP1 mutants infected with HSV1 (KOS) or HSV1(∆ICP6).
